# PAK4 inhibition augments anti-tumour effect by immunomodulation in oral squamous cell carcinoma

**DOI:** 10.1038/s41598-024-64126-0

**Published:** 2024-06-18

**Authors:** Danki Takatsuka, Hidetake Tachinami, Nihei Suzuki, Manabu Yamazaki, Amirmoezz Yonesi, Mayu Takaichi, Shuichi Imaue, Shin-Ichi Yamada, Jun-Ichi Tanuma, Makoto Noguchi, Kei Tomihara

**Affiliations:** 1 Department of Oral and Maxillofacial Surgery, Faculty of Medicine, Academic Assembly, University of Toyama, Toyama, 930-0194 Japan; 2 Life Science Research Center, University of Toyama, Toyama, 930-0194 Japan; 3 Divisions of Oral Pathology, Faculty of Dentistry and Graduate School of Medical and Dental Sciences, Niigata University, Niigata, 951-8514 Japan; 4 Divisions of Oral and Maxillofacial Surgery, Faculty of Dentistry and Graduate School of Medical and Dental Sciences, Niigata University, Niigata, 951-8514 Japan

**Keywords:** Cancer therapy, Oral cancer, Tumour immunology, Cancer

## Abstract

Oral squamous cell carcinoma (OSCC) is one of the most common malignant tumours, warranting novel treatments. Here, we examined the therapeutic efficacy of inhibiting p21 activated kinase 4 (PAK4) in OSCC and determined its immunomodulatory effect by focusing on the enhancement of anti-tumour effects. We examined PAK4 expression in OSCC cells and human clinical samples and analysed the proliferation and apoptosis of OSCC cells following PAK4 inhibition in vitro. We also investigated the effects of in vivo administration of a PAK4 inhibitor on immune cell distribution and T-cell immune responses in OSCC tumour-bearing mice. PAK4 was detected in all OSCC cells and OSCC tissue samples. PAK4 inhibitor reduced the proliferation of OSCC cells and induced apoptosis. PAK4 inhibitor significantly attenuated tumour growth in mouse and was associated with increased proportions of IFN-γ-producing CD8^+^ T-cells. Furthermore, PAK4 inhibitor increased the number of dendritic cells (DCs) and up-regulated the surface expression of various lymphocyte co-stimulatory molecules, including MHC-class I molecules, CD80, CD83, CD86, and CD40. These DCs augmented CD8^+^ T-cell activation upon co-culture. Our results suggest that PAK4 inhibition in OSCC can have direct anti-tumour and immunomodulatory effects, which might benefit the treatment of this malignancy.

## Introduction

Around 90% of all oral cancers are attributed to oral squamous cell carcinoma (OSCC), making it one of the most prevalent malignant tumours globally^1^. Treatment choices involve single-modality surgery, systemic chemotherapy, radiotherapy, or a combination of these methods^2^. For advanced cases, combination therapy is often indicated to improve outcomes and preserve function. Despite notable progress in the diagnosis and treatment of oral cancer, the unresolved challenges lie in its high metastatic potential and resistance to chemotherapy and radiotherapy, posing significant hurdles to patient outcomes^3^.

In recent times, successful clinical strategies focusing on immune checkpoint molecules like CTLA4, PD-1, and PD-L1 have improved outcomes in patients dealing with recurrent or metastatic cancer. Unfortunately, the common occurrence of primary or acquired resistance to these immunotherapies remains a challenge, and effective options to overcome this resistance are currently unavailable^4^. Therefore, novel strategies targeting hitherto untargeted molecules are needed. One particularly intriguing among these approaches is p21 activated kinase 4 (PAK4), widely expressed in different cancers, and it holds significant promise as a potential chemotherapeutic target.

Belonging to the group-II p21 protein (Cdc42/Rac) activated kinase (PAK) family of serine/threonine kinases, PAK4 is instrumental in various biological functions, including cytoskeleton reorganization^5^, cell proliferation^6^, cell migration^7,8^, and cell protection from apoptosis^9^. In multiple cancers, including head and neck cancer, PAK4 is commonly expressed and linked to disease aggressiveness, often correlating with a poor prognosis^10–14^. Targeting of PAK4 was reported to inhibit the WNT/β-catenin pathway and enhanced the anti-tumour effects of PD-1 blockade immunotherapy in melanoma^15^. However, nothing is known about the anti-tumour and immunomodulatory effects of PAK4 inhibition in OSCC.

In this study, we investigated the involvement of PAK4 signaling in the advancement of OSCC. This involved assessing PAK4 expression in human OSCC cell lines and clinical samples. Additionally, we explored the immunomodulatory effects of inhibiting PAK4 in OSCC, using cell lines and a murine OSCC model.

## Materials and methods

### Tissue samples

The research involved 41 patients who underwent surgical intervention for OSCC at the Department of Oral and Maxillofacial Surgery at the University of Toyama Hospital between April 2007 to March 2020. All tissue specimens had not undergone preoperative chemotherapy and were obtained via surgical resection. Nine patients were diagnosed with stage I primary OSCC, nine with stage II, ten with stage III, and thirteen with stage IV OSCC based on the UICC TNM classification criteria for oral cavity cancer^16^. Primary carcinoma sites were the tongue (*n* = 16), mandibular gingiva (*n* = 11), maxillary gingiva (*n* = 6), floor of the mouth (*n* = 4), and buccal mucosa (*n* = 4). Histopathologically, the carcinoma was classified as well-differentiated (*n* = 12), moderately differentiated (*n* = 16), or poorly differentiated (*n* = 13), based on the WHO classification^17^. Of the 41 cases, 24 were male and 17 were female, with a median age of 66 years (20–92 years). The clinicopathological characteristics of the patients are presented in Table 1. The median follow-up period for the surviving patients with OSCCs was 87.5 months (ranging from 18 to 197 months). This study was approved by the ethics committee of the Toyama University Hospital (R2022002).

**Table 1:** Correlation between PAK4 immunoreactivity and clinicopathological variables in 41 patients with oral squamous cell carcinoma.

	n	Median (Interquartile range)	*P* values
Age, years			0.08
< 65	19	74.50 (24.00–94.20)	
≧ 65	22	88.35 (70.08–106.48)	
Sex			0.21
Male	24	86.20 (64.23–109.50)	
Female	17	74.50 (63.60–93.05)	
Primary site			0.062
Tongue	16	68.05 (39.80–85.63)	
Mandibular gingiva	11	91.90 (65.40–104.00)	
Maxillary gingiva	6	94.70 (79.83–143.20)	
Buccal mucosa	4	92.50 (61.95–114.50)	
Floor of mouth	4	90.90 (57.80–107.58)	
Tumour stage			0.28
I	9	74.50(49.35–85.55)	
II	9	87.50(50.80–94.25)	
III	10	80.20(59.75–107.63)	
IV	13	89.20(75.75–115.95)	
TNM classification			
T category			0.018
T1	13	65.40(41.35–85.55)	
T2	11	79.40(63.70–94.20)	
T3	6	80.20(59.98–143.18)	
T4	11	100.20(88.40–119.90)	
N category			0.34
N0	23	85.70(61.60–94.30)	
N1	8	68.85(51.85–91.23)	
N2	10	93.60(69.95–126.33)	
Differentiation			0.41
Well	12	69.95(46.18–99.73)	
Moderetion	16	83.2(65.65–93.70)	
Poorly	13	87.50(63.95–123.80)	
Smoking			0.75
Absence	21	85.40(55.80–107.00)	
Presence	20	85.45(63.63–93.63)	
Drinking			0.83
Absence	21	81.00(55.80–101.80)	
Presence	20	86.20(63.63–97.28)	

### Cell lines

Human OSCC cell lines, HSC-2, HSC-3, HSC-4, and Ca 9–22, were obtained from the RIKEN Bio Resource Center Cell Bank. The cells were cultured in RPMI 1640 medium supplemented with 10% foetal bovine serum (FBS) containing 1% penicillin–streptomycin and 1% sodium pyruvate in a humidified incubator set at 5% CO_2_.

The mouse OSCC cell line SCCVII is an established cell line that spontaneously arose from the C3H/HeN mouse strain. The mouse OSCC cell line NR-S1K was kindly provided by Dr. M. Azuma from the Department of Molecular Immunology, Graduate School, Tokyo Medical and Dental University. This cell line was derived from the NR-S1 cell line, which, in turn, was established from C3H/HeN mice^18^. The cells were maintained in RPMI1640 medium, supplemented with 10% FBS.

### Mice and tumour model

The tumour model was constructed as previously described^19^. Female C3H/HeN mice (8-weeks-old) were purchased from Sankyo Labo Service Corporation, Inc. and housed under specific pathogen-free conditions according to the institutional guidelines prescribed by the University of Toyama. The animal protocols were reviewed and approved by the Institutional Animal Care and Use Committee of the University of Toyama (A2022MED-31).

NR-S1K cells (1 × 10^6^) were subcutaneously injected into the right masseter of C3H/HeN mice. After seven days of tumours inoculation, when tumour surface areas reached around 20mm^2^, the tumour area (length × width) was measured daily using a calliper (Fig. 4a, Supplementary Fig. S1).

### Immunohistochemistry

Immunohistochemistry was performed with reference to methods previously described^20^. De-paraffinised and rehydrated sections were immersed in 10 mM citric acid buffer (pH 6.0) and heated for 10 min at 121 °C for antigen retrieval. For blocking the endogenous peroxidase activity, the sections were immersed in 0.3% hydrogen peroxide in methanol for 15 min at room temperature (range of 15–25 °C). The sections were immersed in Blocking I (Nakarai Tesque, Kyoto, Japan) for 10 min at room temperature to block non-specific proteins, and then incubated overnight with the primary antibody (anti-PAK4 mouse monoclonal antibody 1:500; clone B-3; Santa Cruz Biotechnology, Texas, USA) at 4 °C. The sections were then incubated with a peroxidase-conjugated secondary antibody (anti-rabbit polyclonal antibody; #424,144; Nichirei Biosciences, Tokyo, Japan) for 30 min at room temperature. Peroxidase reaction was performed using 3,3′-diaminobenzidine substrate solution (Dako) as a substrate and the sections were counterstained with haematoxylin.

The staining intensity for each sample was designated as follows: no expression (0), weak (1 +), moderate (2 +), and strong (3 +) (Supplementary Fig. S2). In addition, the images of HE-stained and immunostained specimens were captured using an Aperio CS2 scanner (Leica, Tokyo, Japan) and a virtual slide was created; the expression in each immunostained specimen was analysed using an Aperio Image Scope analyser (Leica, Tokyo, Japan). The invasion sites were confirmed in the HE specimen and the hot spot area, which represented the zone with the highest number of PAK-positive stained cells among 2000–3000 cancer cells, was analysed. The percentage of cells at each staining intensity level was calculated and the H-score was derived using the following formula: H-score = (1 × % cells 1 +) + (2 × % cells 2 +) + (3 × % cells 3 +)^21^.

Furthermore, the correlation between the H-scores and clinicopathological factors was analysed using bivariate analysis. The clinicopathological factors included patient sex and age, primary site, histopathological differentiation, tumour stage, TNM classification, and smoking and drinking (Table 1). Additionally, the patient's overall survival prognosis was compared by categorizing PAK4 expression as either positive (≧ 80 of H-score) or negative (< 80 of H-score).

### Assessment of PAK4 expression in OSCC cells

Intracellular PAK4 expression in the human OSCC cell lines, HSC-2, HSC-3, HSC-4, and Ca 9–22, was detected using flow cytometry (FACS Celesta, Becton Dickinson). The cells were fixed with 4% paraformaldehyde/PBS for 10 min at 37 °C and then pre-chilled on ice for 1 min followed by permeabilization with 90% ice-cold methanol (Nakarai Tesque, Kyoto, Japan) for 30 min. The cells were then stained with an anti-PAK4 pAb (FITC-conjugated human pAb, Abcam). The mean fluorescence intensity ratio was analysed using the FlowJo software (FlowJo, LLC, Ashland, OR, USA).

### Cell proliferation assay

Cell proliferation was assessed as previously described^22^. Human and mouse OSCC cell lines were seeded in 96-well plates at a density of 1.5 × 10^4^ cells/well. The PAK4 inhibitor, PF-3758309 (Pfizer, New York, USA), was added to the wells and the cells were cultured for 48 h. The concentrations of PF-3758309 were 0 (control), 0.5, 1.0, 2.5, and 5.0 μM, respectively. The viability of the adherent cells was measured by adding tetrazolium salt and 4-[3-(4-iodophenyl)-2-(4-nitrophenyl)-2H-5-tetrazolio]-1,3-benzene disulfonate (WST-1) premix (Takara Bio) to each well. The cleavage of WST-1 into formazan by metabolically active cells was measured by scanning the plates at 450 nm using a microtiter plate reader (Filter Max F5; Molecular Devices, Tokyo, Japan).

### Assessment of cellular apoptosis

Apoptotic cell death was assessed as previously described^23^. Human and mouse OSCC cell lines were cultured in the presence of PF-3758309 (2.5 μM) for 48 h. Apoptosis was assessed using a Fluorescein Isothiocyanate (FITC) Annexin V Apoptosis Detection Kit II (BD Pharmingen, San Diego, CA, USA) according to the manufacturer’s instructions. The cells were stained with FITC-conjugated annexin V and propidium iodide (PI) for 15 min at room temperature in the dark before analysis using flow cytometry (FACS Celesta, Becton Dickinson). The proportion of apoptotic cells was calculated as the percentage of annexin V-positive cells. Annexin V-positive/PI-negative cells were considered early apoptotic cells, whereas annexin V-positive/PI-positive cells were considered late apoptotic cells^23^.

### In vivo* PAK4 inhibitor treatment*

PAK4 inhibitor treatment to mice was performed with reference to methods previously described. Seven days after inoculation of NR-S1K, mice were injected intraperitoneally (i.p.) with 25 mg/kg PF-3758309 at 2-day intervals (Fig. 4a). Twelve days after the administration of PF-3758309, mice were sacrificed, and cells from tumours, peripheral blood, spleen, and lymph nodes were analysed using flow cytometry (FACS Celesta, Becton Dickinson). DNase I (0.02 mg/mL; Roche, Switzerland) and collagenase type IV (1 mg/mL; Sigma–Aldrich, St Louis, MO) were used to digest the harvested tumours at 37 °C for 60 min for dissociation of tumour cells. Single cell suspension was generated by filtration through a 100 μm nylon cell strainer (FALCON^™^).

### Antibodies

The following antibodies were obtained from Thermo Fisher Scientific: FITC-conjugated antibodies against mouse CD11b, CD40, and CD83; PE-conjugated antibodies against mouse IFN-γ, CD11c, β-catenin, CD274, CD80, MHC class I, and MHC class II; PerCP-Cy5.5-conjugated antibodies against mouse CD4; PE-Cy7-conjugated antibodies against mouse CD8 and CD11c; APC-conjugated antibodies against mouse FoxP3, Ly-6G(Gr-1), and CD86; and APC-Cy7-conjugated antibodies against mouse CD3e, and CD103.

### Flow cytometry

The samples were blocked with purified FcR-blocking mAb (Thermo Fisher Scientific), washed, and suspended in PBS supplemented with 2% FBS, 0.05% sodium azide, and a saturating concentration of fluorochrome-conjugated mAbs. The cells were analysed using a FACS Celesta (Becton Dickinson, San Jose, CA, USA).

### T-cell proliferation assay and intracellular cytokine staining

Splenic CD3^+^ T-cells were isolated from naïve C3H/HeN mice by cell sorting using FACS Aria SORP (Becton Dickinson, San Jose, CA, USA) and labeled with carboxyfluorescein succinimidyl ester (CFSE; Invitrogen). Dendritic cells (DCs; CD11c^+^CD103^−^ cells and CD11c^+^CD103^+^ cells) were purified from the tumours of OSCC tumour-bearing mice after administration of PF-3758309 by cell sorting using FACS Aria SORP. CFSE-labelled T-cells were stimulated with DCs in the presence of 0.1 μg/mL of an anti-CD3 Ab (eBioscience) for 72 h at a T cell : DC ratio of 10 : 3. Four hours before the end of culture, 50 ng/mL of phorbol 12-myristate 13-acetate (Sigma–Aldrich), 500 ng/mL of ionomycin (Sigma–Aldrich), and 4 μM of monensin (eBioscience) were added to the culture. After staining for surface antigens, the cells were fixed, permeabilised, and used for intracellular staining of IFN-γ. The CFSE fluorescent dye and the IFN-γ production were analysed using flow cytometry. This co-culture method was based on previously described studies^24^.

### Statistical analysis

Groups were compared using the Wilcoxon rank sum test or Kruskal–Wallis test with the statistical software Using JMP Pro17.0.0 for Mac (SAS Institute). For the survival analysis, Kaplan–Meier survival curves were constructed based on univariate predictors, and differences between groups were assessed using the log-rank test. A value of *p* < 0.05 was considered statistically significant.

## Results

### PAK4 was expressed in OSCC cell lines and patients’ tissue samples

**Figure 1:**
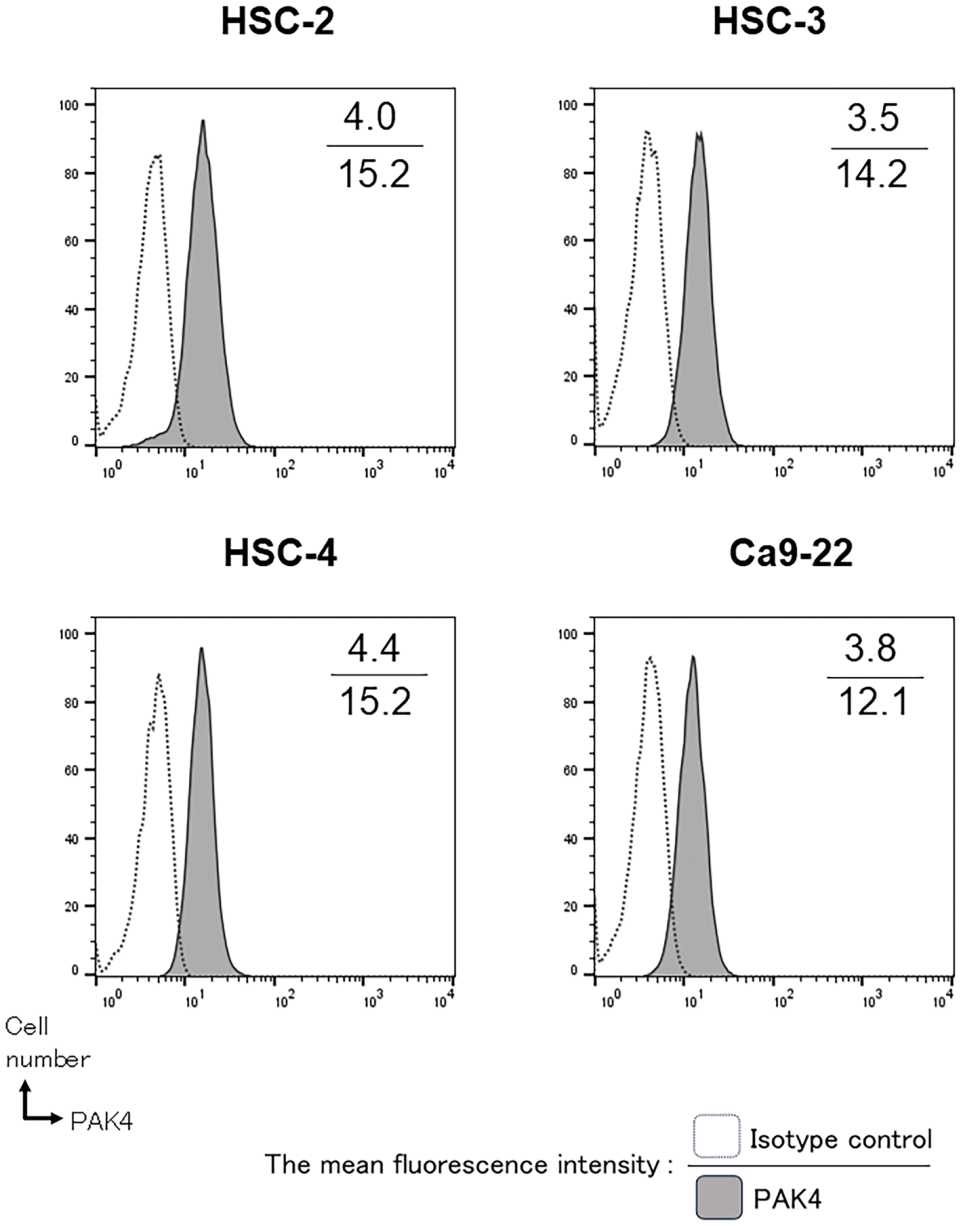
Expression of PAK4 in oral squamous cell carcinoma (OSCC) cell lines. Intracellular staining of PAK4 in the human OSCC cell lines, HSC-2, HSC-3, HSC-4, and Ca9-22, was performed using flow cytometry. Representative histograms indicate PAK4 expression in each cell line (*n* = 3/group). Numbers in each panel indicate the mean fluorescence intensity for the control (upper value) and anti-PAK4 (lower value) antibodies.

Intracellular expression of PAK4 in OSCC cell lines was analysed using flow cytometry. The expression of PAK4 was confirmed in all the human OSCC cell lines (Fig. 1).

**Figure 2:**
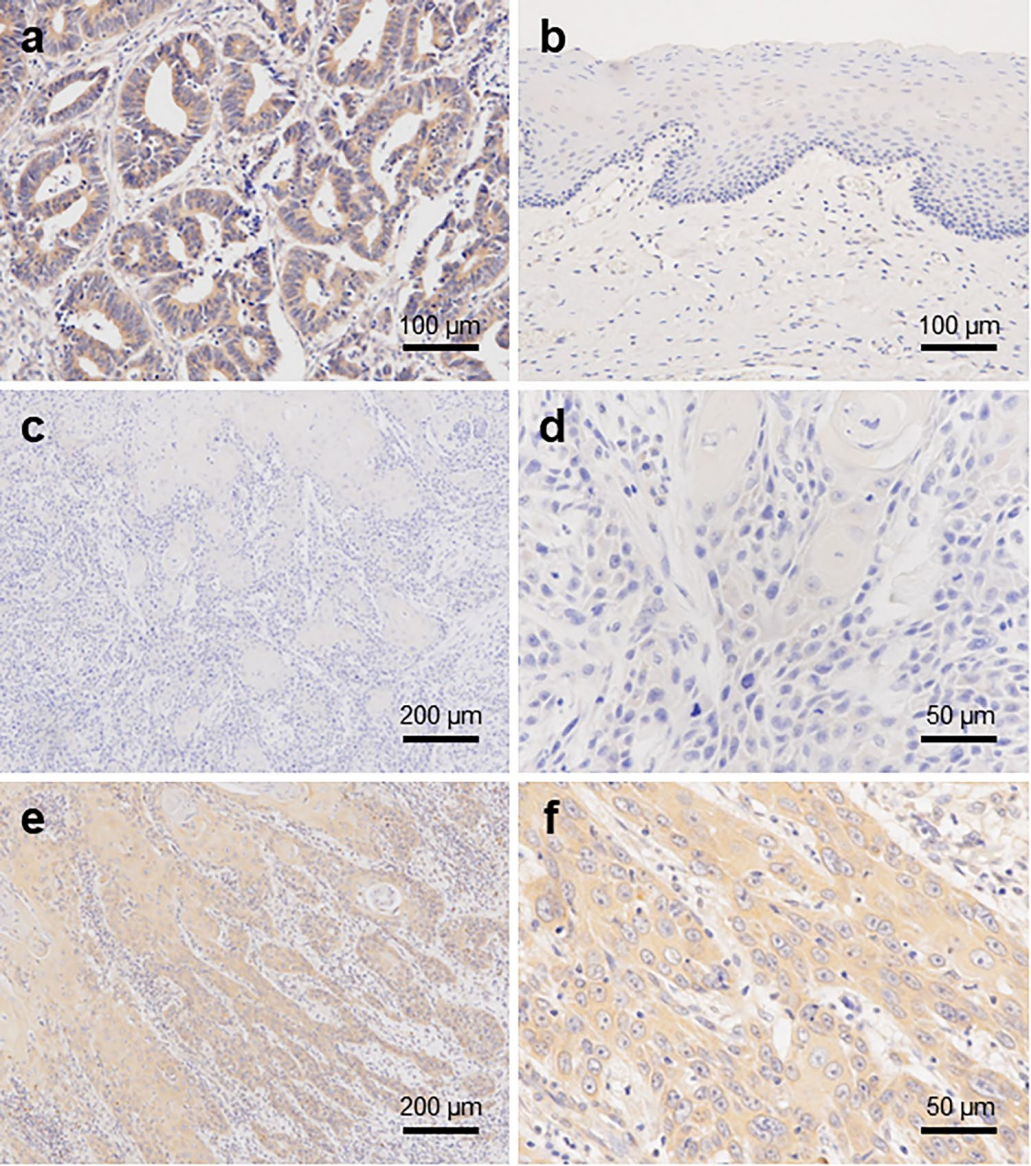
Immunohistochemical assessment of PAK4 expression in oral squamous cell carcinoma (OSCC) cases. Immunohistochemical staining profile for PAK4 in colorectal carcinoma tissue [positive control] (**a**), normal oral epithelia and OSCC tissues: normal oral epithelia (**b**), well-differentiated OSCC (**c**, **d**), and poorly differentiated OSCC (**e**, **f**).

In all OSCC patient tissue samples, immunohistochemical analysis revealed PAK4 expression in the cytoplasm, which was contrasted with the positive control colorectal carcinoma tissue (Fig. 2a). The protein expression was not detected in the normal epithelium (Fig. 2b). In well-differentiated carcinoma cells, the immunostaining of PAK4 was slightly more intense than that in the normal epithelium and was mainly observed in the cytoplasm in the carcinoma areas (Fig. 2c,d). In contrast, strong PAK4 staining was observed in poorly differentiated carcinoma cells (Fig. 2e,f). The immunoreactivity of PAK4 in each sample was compared using staining intensity. Among the various clinicopathological characteristics, PAK4 expression was significantly associated with category T according to the TNM classification (Table 1). The staining intensity of PAK4 showed a significant association with more advanced category T (*P* = 0.018) and was notably elevated in patients with larger primary tumour volumes. However, the association of PAK4-staining intensity was not significantly different from that of other characteristics, including age and sex of patients, location of tumours, differentiation, category N, stage in the TNM classification, smoking, or drinking. Despite patients with positive PAK4 expression in tumours showing a tendency toward a poorer prognosis, the Although the Kaplan–Meier survival analysis revealed no statistically significant association between PAK4 immunoreactivity in tumour cells and overall survival across all cases, patients with positive PAK4 expression in their tumours tended to have a poorer prognosis (*P* = 0.094, log-rank test, Supplementary Fig. S3).

### PAK4 was involved in the growth and survival of OSCC cells

**Figure 3:**
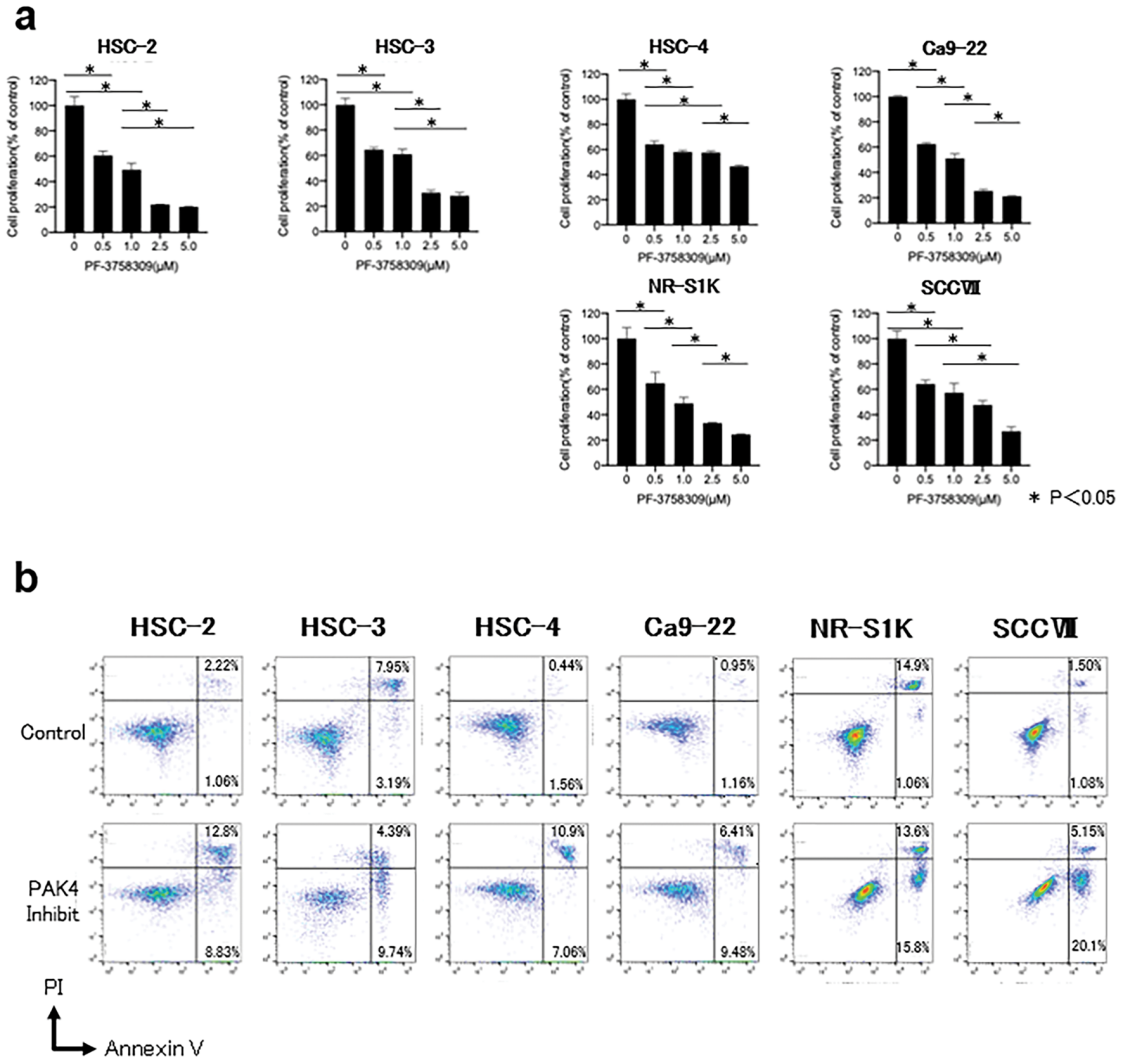
Growth inhibition and apoptosis induction following administration of PAK4 inhibitor in oral squamous cell carcinoma (OSCC) cell lines. Human OSCC cell lines, HSC-2, HSC-3, HSC-4, and Ca9-22, and mouse OSCC cell lines, SCCVII and NR-S1K, were cultured in PAK4 inhibitor, PF-3758309, for 48 h. (**a**) The proliferative activity of cells was measured using the WST-1 cell proliferation assay. The asterisks indicate a significant difference between two groups. (**b**) The cells were stained with FITC-conjugated annexin V and propidium iodide (PI). The proportion of apoptotic cells was calculated as the percentage of annexin V-positive cells. Annexin V-positive/PI-negative cells were considered early apoptotic, whereas annexin V-positive/PI-positive cells were considered late apoptotic cells.

To investigate whether PAK4 is involved in the growth and survival of OSCC cells, alterations in the viability of OSCC cells upon selective inhibition of PAK4 were assessed. The OSCC cell viability was significantly reduced following treatment with PF-3758309, the PAK4 inhibitor (Fig. 3a). Next, we examined whether PAK4 inhibition affected the apoptosis of OSCC cells. The number of apoptotic cells increased significantly following PF-3758309 treatment (Fig. 3b).

Overall, these data provide strong evidence supporting the contribution of PAK4 signalling to the growth and survival of OSCC cells and to the anti-tumour efficacy of PAK4 inhibitor via modulation of these cellular processes.

### *PAK4 inhibition modulated the distribution of immune cell populations in *in vivo* mouse OSCC model*

**Figure 4:**
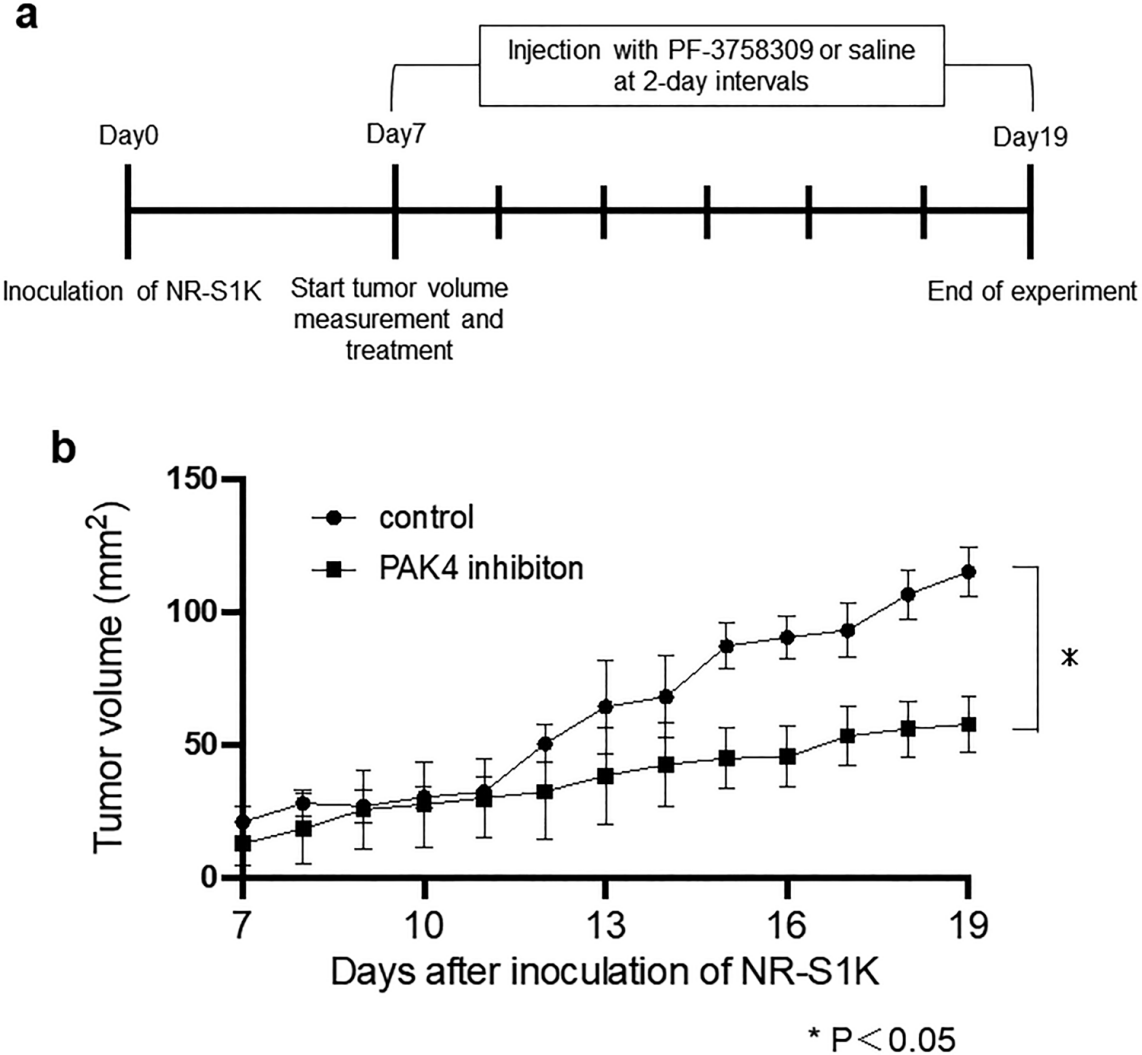
Growth curve of subcutaneous tumours in PAK4 inhibitor-treated and control mice. (**a**) NR-S1K cells (1 × 10^6^) were subcutaneously injected into the right masseter of C3H/HeN mice. On day 7 after tumour induction, when the tumour had grown to around 20mm^2^, the tumour area (length × width) was measured daily, and treatment with injection of PF-3758309 or saline was administered at 2-day intervals. (**b**) The tumour size in the mice was measured at the indicated time points (*n* = 5/group). *p < 0.05, control vs. PF-3758309.

**Figure 5:**
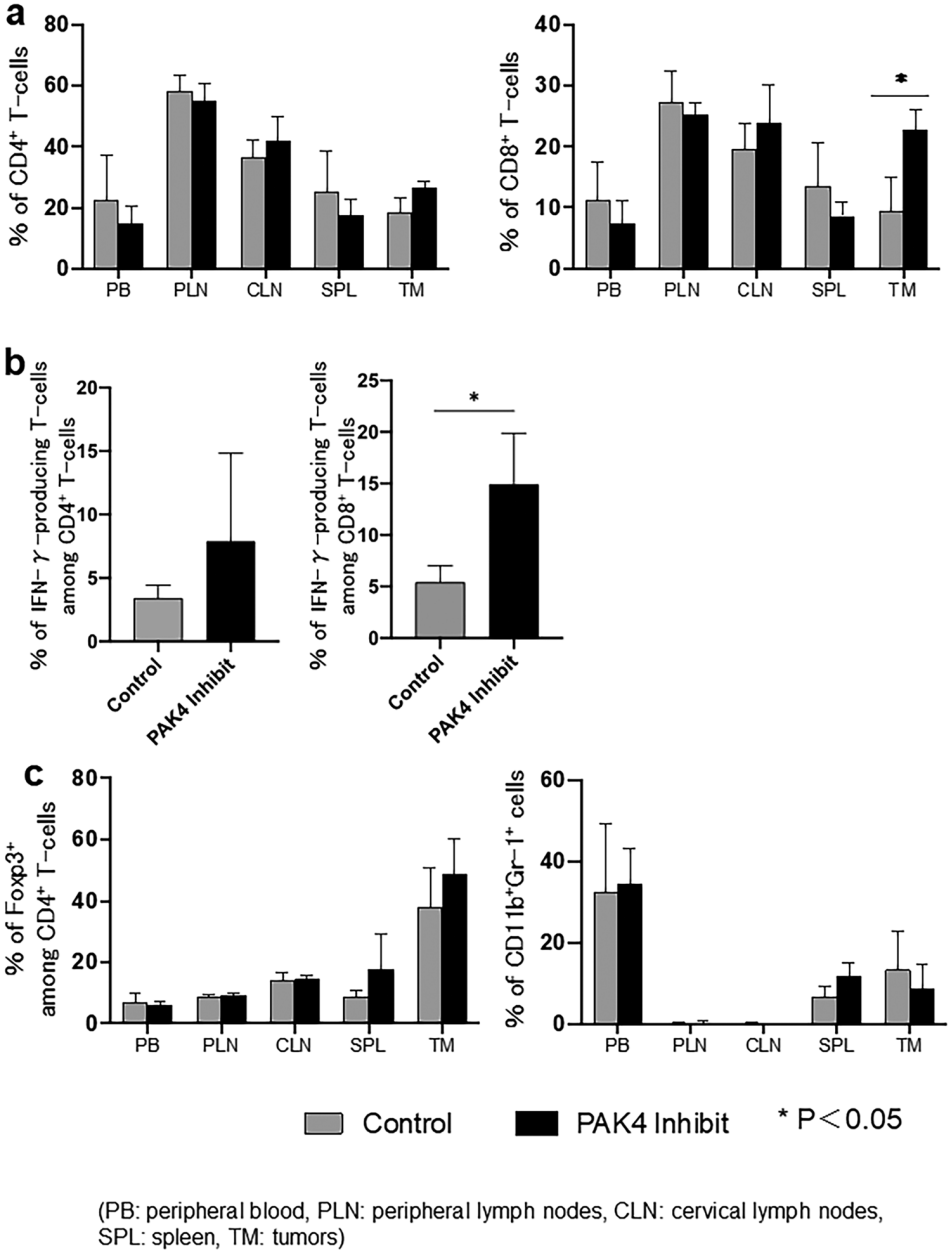
Proportion of immune cells following in vivo administration of PAK4 inhibitor in oral squamous cell carcinoma (OSCC) tumour-bearing mice. After 12 days of treatment with the PAK4 inhibitor, tumours (TM), peripheral blood (PB), cervical lymph nodes (CLN), peripheral lymph nodes (PLN), and spleen (SPL) were harvested, and the percentages of different immune cell types in each of the samples from control and PAK4 inhibitor-treated mice were determined using flow cytometry. A summary of these results is shown (*n* = 5/group); *p* < 0.05, control vs PF-3758309. (**a**) CD4^+^ and CD8^+^ T-cells, (**b**) IFN-γ-producing T-cells among CD8^+^ and CD4^+^ T-cells, (**c**) CD4^+^Foxp3^+^ regulatory T-cells (Tregs) and CD11b^+^Gr-1^+^ myeloid-derived suppressor cells (MDSCs), Experiments were performed in triplicate and similar results were obtained.

To investigate whether PAK4 inhibition could affect the distribution of immune cells in vivo, we examined alterations in the proportion of different immune cells in OSCC tumour-bearing mice following the administration of the PAK4 inhibitor. PAK4 inhibitor-treated mice showed significantly delayed tumour growth compared with control mice (Fig. 4b). The proportion of CD8^+^ T-cells was significantly increased in the tumours, but not in the peripheral blood, cervical lymph nodes, peripheral lymph nodes, and spleen of PAK4 inhibitor-treated mice compared with that in the respective tissue of control mice (Fig. 5a). In contrast, the proportion of CD4^+^ T-cells was not altered between the PAK4 inhibitor-treated and control mice (Fig. 5a). We then evaluated the proportion of IFN-γ-producing T-cells in the tumours. The proportion of IFN-γ-producing T-cells among CD8^+^ T-cells was significantly increased in the tumours of PAK4 inhibitor-treated mice compared with that in control mice (Fig. 5b). The proportions of regulatory T-cells (Tregs) and myeloid-derived suppressor cells (MDSCs) did not differ between PAK4 inhibitor-treated and control mice (Fig. 5c).

These results suggest that treatment with the PAK4 inhibitor enhances the anti-tumour immune response of T-cells in tumours.

### PAK4 inhibition altered the expression of β-catenin in OSCC cells

To investigate whether PAK4 inhibition could affect the Wnt/β-catenin signalling, we compared the expression status of β-catenin in OSCC cells in the presence or absence of PAK4 inhibitor.

**Figure 6:**
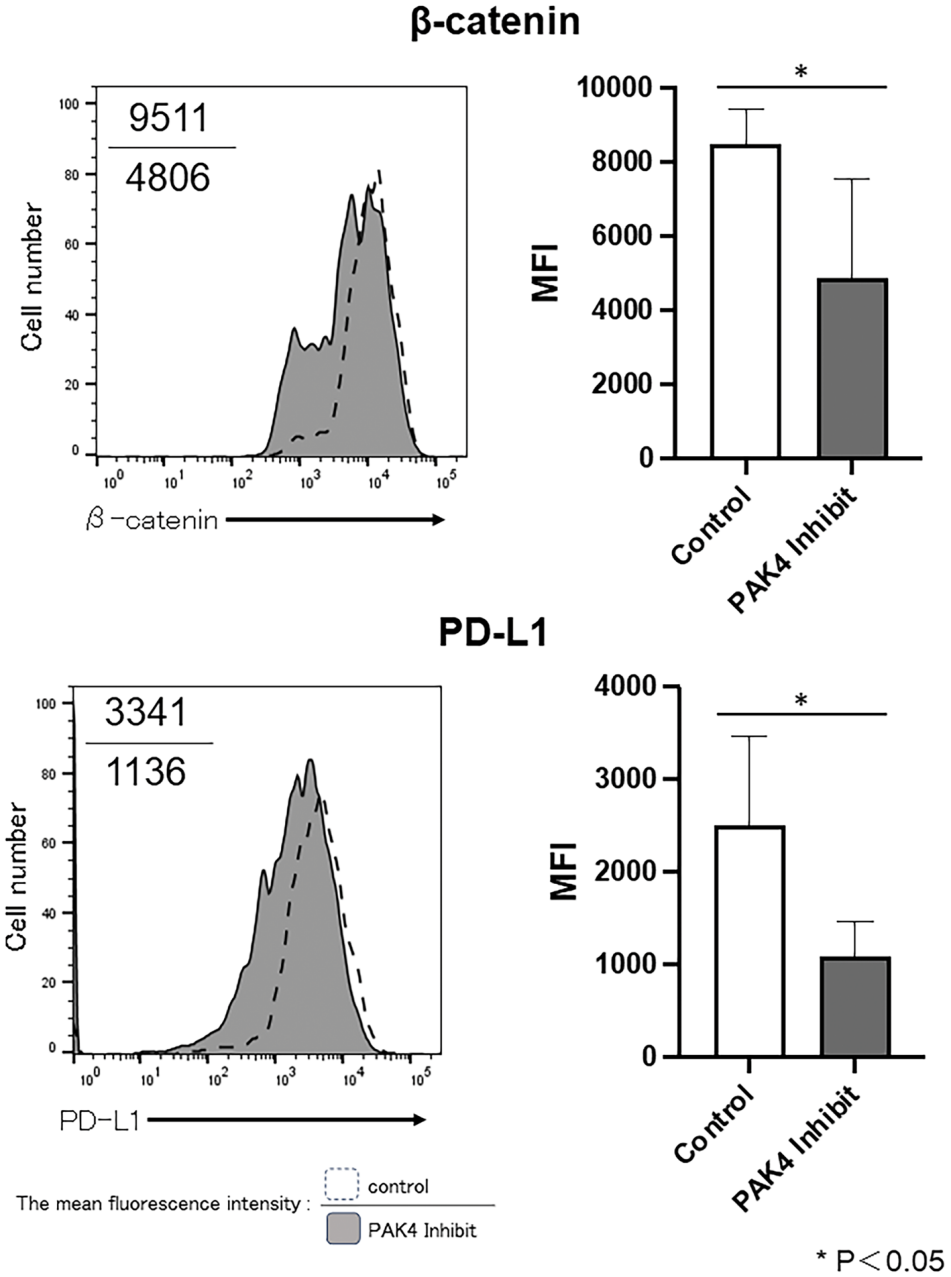
Expression of β-catenin and PD-L1 following administration of PAK4 inhibitor in oral squamous cell carcinoma (OSCC) tumour-bearing mice. In OSCC tumour-bearing mice administered the PAK4 inhibitor, PF-3758309, the expression of β-catenin in tumour cells and of PD-L1 on the surface of tumour cells was analysed using flow cytometry. Representative histograms and bar graph of overall results are shown (n = 4/group); *p* < 0.05, control versus PF-3758309. Numbers in each panel indicate the mean fluorescence intensity of each molecule in control (upper value) and PAK4 inhibitor-treated (lower value) mice.

The expression of β-catenin was abrogated in OSCC cells after treatment with PF-3758309 (Supplementary Fig. S4). Moreover, the expression of β-catenin in tumour cells of the OSCC tumour-bearing mice, with or without PAK4 inhibitor treatment, was also compared. The expression of β-catenin was significantly lower in tumour cells of PAK4 inhibitor-treated mice than that that in control mice (Fig. 6).

These results suggest that PAK4 is involved in the Wnt/β-catenin signalling and regulates the expression of β-catenin.

### PAK4 inhibition modulated the function of T-cells and dendritic cells in OSCC tumour-bearing mice

Recently, the anti-tumour immune effects have been demonstrated to be caused by the activation of the Wnt/β-catenin pathway^15^. The activation of the Wnt/β-catenin pathway induces PD-L1 expression on tumour cells, which could lead to the inactivation of T-cells^25^. Furthermore, the activation of the Wnt/β-catenin pathway suppresses the cross-priming of DCs to CD8^+^ T-cells^15^. Therefore, we analysed the expression of PD-L1 on tumour cells following PAK4 inhibition in OSCC tumour-bearing mice and determined the function of DCs within tumour tissues. The expression of PD-L1 was found to be significantly lower on tumour cells of PAK4 inhibitor-treated mice than in control mice (Fig. 6).

**Figure 7:**
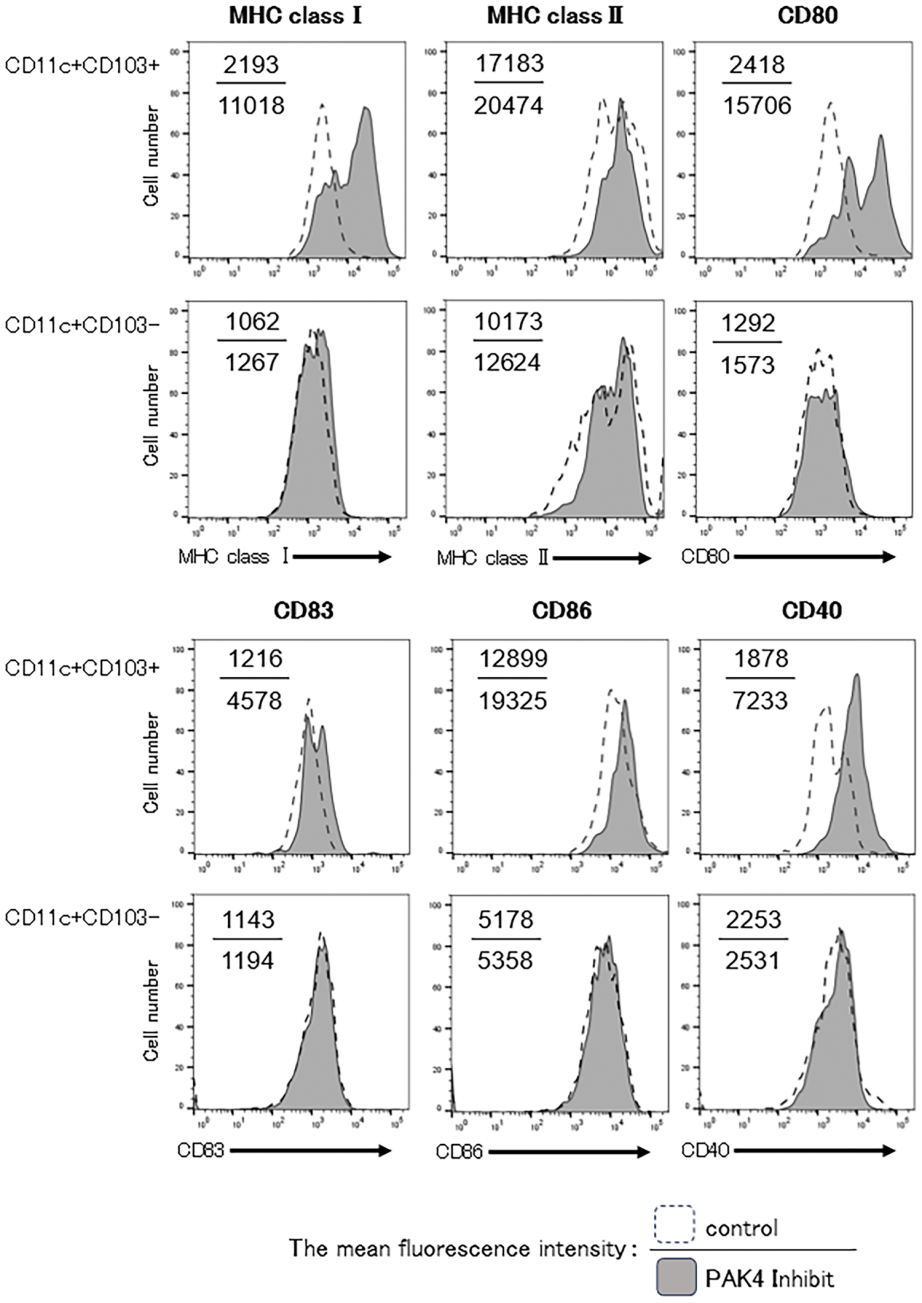
Phenotypic alteration of tumour-infiltrated dendritic cells (DCs) following administration of PAK4 inhibitor in oral squamous cell carcinoma (OSCC) tumour-bearing mice. The phenotypic profiles of DCs in OSCC tumour-bearing mice after administration of the PAK4 inhibitor were analysed. Cells were harvested from the tumours of control or PAK4 inhibitor-treated mice, and the cell-surface expression of various lymphocyte co-stimulatory molecules on CD11c^+^CD103^−^ and CD11c^+^CD103^+^ cells was determined using flow cytometry. Representative histograms are shown (*n* = 4/group). Numbers in each panel indicate the mean fluorescence intensity of each molecule in control (upper value) and PAK4 inhibitor-treated (lower value) mice.

As β-catenin inhibits CCL4 production, which is important for the regulation of CD103^+^ DCs, we evaluated the effect on the function of CD11c^+^CD103^+^ DCs due to the abrogation of β-catenin via PAK4 inhibition. We compared the surface phenotypes of tumour-infiltrating CD11c^+^CD103^+^ DCs between PAK4 inhibitor-treated and control mice. While various lymphocyte co-stimulatory molecules, such as major histocompatibility complex (MHC) class I and II molecules, CD83, CD86, and CD40 on CD11c^+^CD103^+^ DCs within tumours of mice treated with PAK4 inhibitors, did not show statistically significant differences compared to control mice, there were trend toward increased expression levels. Additionally, it was found that the expression level of CD80 on CD11c^+^CD103^+^ DCs in the tumours of mice treated with PAK4 inhibitors was significantly higher. As for the molecular groups on CD11c^+^CD103^-^ DCs, no notable differences were observed between PAK4 inhibitor-treated mice and control mice (Fig. 7, Supplementary Fig. S5).

**Figure 8:**
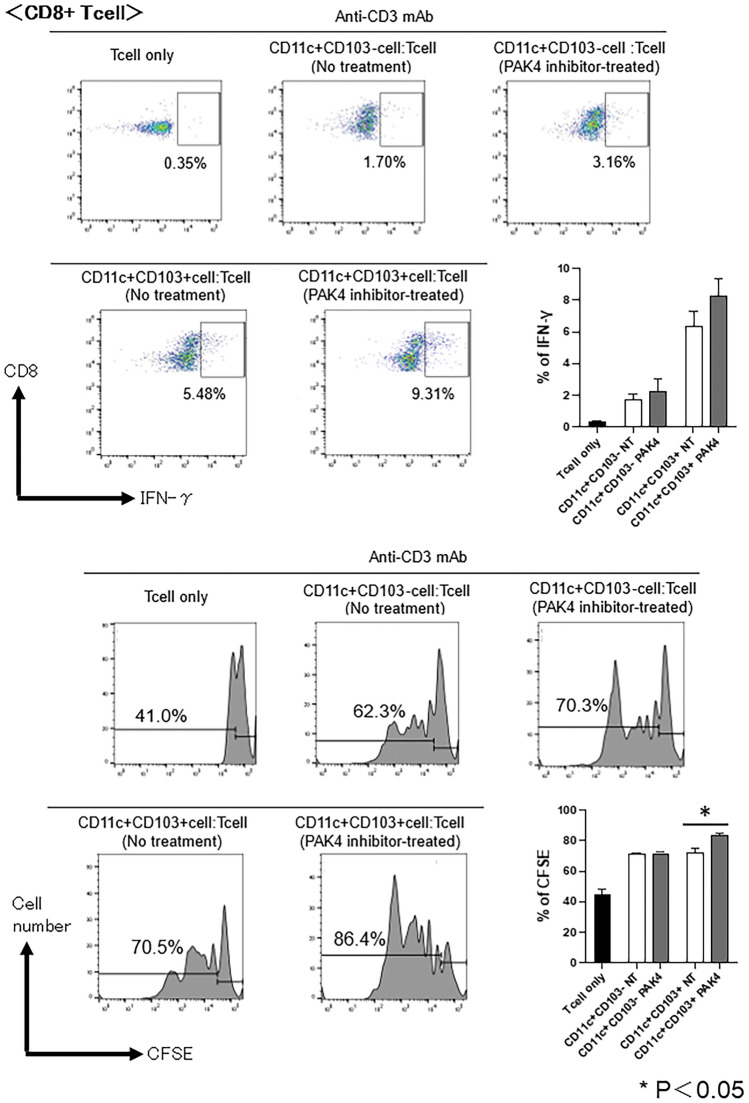
T-cell stimulatory capacity of dendritic cells (DCs) following administration of PAK4 inhibitor in oral squamous cell carcinoma (OSCC) tumour-bearing mice. Splenic CD3^+^ T-cells were isolated from naïve C3H/HeN mice and labelled with carboxyfluorescein succinimidyl ester (CFSE). DCs (CD11c^+^CD103^−^ cells and CD11c^+^CD103^+^ cells) were sorted from the tumours of OSCC tumour-bearing mice. CFSE-labelled T-cells were co-cultured with DCs in the presence of 0.1 μg/mL of an anti-CD3 Ab for 72 h at a T cell:DC ratio of 10:3. The CFSE fluorescent dye and the IFN-γ-producing T-cells among CD8^+^ T-cells were analysed using flow cytometry. Representative histograms and bar graphs of overall results are shown; *p* < 0.05, control vs PF-3758309.

To evaluate the function of DCs, we compared the stimulatory functions of CD11c^+^CD103^+^ DCs and CD11c^+^CD103^−^ DCs from the tumours of PAK4 inhibitor-treated mice by co-cultivation with splenic T-cells from naive mice in vitro. DCs from PAK4 inhibitor-treated mice exhibited a heightened stimulatory effect on CD8^+^ T-cell proliferation and the proportion of IFN-γ-producing T-cells among CD8 + T-cells in vitro compared to those from control mice. Moreover, CD8^+^ T-cells co-cultured with CD11c^+^CD103^+^ DCs from PAK4 inhibitor-treated mice significantly increased in number. Furthermore, the stimulatory effect of CD11c^+^CD103^+^ DCs from PAK4 inhibitor-treated mice on CD8^+^ T-cells was superior to that of CD11c^+^CD103^−^ DCs (Fig. 8).

These results suggest that PAK4 inhibition in OSCC tumour-bearing mice abrogates the expression of PD-L1 on tumour cells and increases the expression of MHC molecules and lymphocyte co-stimulatory molecules on DCs in tumours, enhancing the anti-tumour immune response. In addition, CD103^+^ DCs, a subset of DCs, may play an important role in promoting the anti-tumour immune responses via PAK4 inhibition.

## Discussion

Immunotherapy using ICIs has shown better clinical efficacy than conventional chemotherapy for various types of tumours. However, the overall response rate in patients with recurrent or metastatic HNSCC is less than 20%^4^. Therefore, novel strategies combined with other agents that target other novel molecules are desired.

The expression of PAK4 increases with disease progression in several types of tumours, including HNSCC, and is associated with the aggressiveness of disease and poor prognosis^10–14^. Targeting of PAK4 has been reported to have growth inhibitory effects in gastric, lung, and pancreatic cancer^26–28^. Furthermore, targeting of PAK4 has immunomodulatory effects through the inhibition of the WNT/β-catenin pathway^15^. However, little is known about the expression of PAK4 and about the anti-tumour and immunomodulatory effects of PAK4 inhibition in OSCC. In the present study, we examined the effects of PAK4 inhibition as a therapeutic target in OSCC and investigated its immunomodulatory effects by focusing on the enhancement of anti-tumour effects.

Activation of PAK4 is induced by Cdc42, a small GTP-binding protein in the Rho family. PAK4 is highly expressed in embryos but not in adult tissues, which is suggestive of its crucial role in embryonic development^29^. However, overexpression of PAK4 in adults may cause the formation and growth of tumours^10^.

In the present study, we confirmed that PAK4 is expressed in all the selected human OSCC cell lines. Moreover, PAK4 expression was immunohistochemically detected in tissue samples from patients with OSCC in the tumour area, but not in the adjacent normal area. Furthermore, the immunohistochemical staining intensity of PAK4 in OSCC cases was found to be positively correlated with tumour size. These results strongly suggested the involvement of PAK4 in the promotion of OSCCs and its potential as a novel therapeutic target.

With regard the molecular mechanism of tumour promotion by PAK4, activation of the PI3K/Akt signalling pathway through Akt phosphorylation^30–32^ and activation of the ERK signalling pathway through MEK phosphorylation are critical^33,34^. Moreover, PAK4 inhibits apoptotic cell death in cancer cells through BAD phosphorylation^35^ and consequently prevents the abrogation of cytochrome C release via the mitochondrial pathway^36^. Furthermore, it has been shown that PAK4 protects the cells from apoptosis by the abrogation of caspase-8 activation^9^.

In the present study, PAK4 inhibition abrogated the proliferation of OSCC cells and increased the apoptotic cell death. These results strongly suggest that PAK4 contributes to the proliferation and survival of OSCC cells and may be a potential target for the treatment of OSCC.

Recently studies have demonstrated the anti-tumour immune effect by targeting PAK4, which could be because of the activation of the Wnt/β-catenin pathway. In the cytoplasm of cancer cells, PAK4 induces the phosphorylation and nuclear translocation of β-catenin^15^. Moreover, activation of the Wnt/β-catenin pathway induces PD-L1 expression in tumour cells, which can lead to the inactivation of T-cells^25^. Furthermore, the activation of the Wnt/β-catenin pathway suppresses the cross-priming of DCs to CD8^+^ T-cells^15^, particularly affecting the function of CD103^+^ DCs that require decrease in CCL4 through the activation of the Wnt/β-catenin pathway^37^. Generally, it is believed that DCs play a central role in antitumour immune responses by mediating the priming and activation of T-cells^37–39^. Conventional dendritic cells (cDCs) are efficient antigen-presenting cells and consist two functional specialized subsets, conventional type 1 dendritic cells (cDC1) and conventional type 2 dendritic cells (cDC2)^40^. CDC1, with CD8α ^+^ and/or CD103^+^ DCs as the main subsets cross-presents tumour antigens to CD8 + T-cells^40,41^. In particular, CD103^+^ DCs play an important role in the immune cycle, and it has been reported that lack CD103^+^ DCs within the TME resist the effector phase of the anti-tumour T-cell response, contributing to immune escape^42–44^.

In the present study, the PAK4 inhibitor abrogated the expression of β-catenin and PD-L1 in tumour cells of the OSCC tumour-bearing mice. In addition, the PAK4 inhibitor increased the expression of MHC and immune co-stimulatory molecules in tumour-infiltrated CD103^+^ DCs. Furthermore, we confirmed the significant CD8^+^T-cell stimulatory function of tumour-infiltrated CD103^+^ DCs in PAK4 inhibitor-treated mice compared with that in non-treated mice via an in vitro T-cell proliferation assay. On the basis of these results, PAK4 targeting could enhance the host anti-tumour immunity via T-cells by reducing PD-L1 on tumour cells and activating DCs, especially CD103 + DCs through suppression of the Wnt/β-catenin pathway in OSCC.

Taken together, our results revealed that the targeting PAK4 in OSCC exerts not only the direct anti-tumour effect such as suppressing tumour cell proliferation but also enhancement of anti-tumour immune responses by activating T cell immune responses.

Thus, PAK4 is a potential novel therapeutic target for OSCC. Targeting of PAK4 may be a novel strategy for combination immunotherapy considering its ability to modulate host immunity in OSCC.

### Supplementary Information

Supplementary Figures.

Supplementary Legends.

## Data Availability

Any personal or patient data are unavailable due to privacy or ethical restrictions. All other data are available from the corresponding author upon reasonable request.
