# Supplementary material for: PAK4 inhibition augments anti-tumour effect by immunomodulation in oral squamous cell carcinoma
Source: Sci Rep. 2024 Jun 18;14:14092. doi: 10.1038/s41598-024-64126-0 (PMC11189426; doi:10.1038/s41598-024-64126-0)
Supplement: Supplementary file 1 — Supplementary Figures. [file 41598_2024_64126_MOESM1_ESM.pptx]

## Slide 1
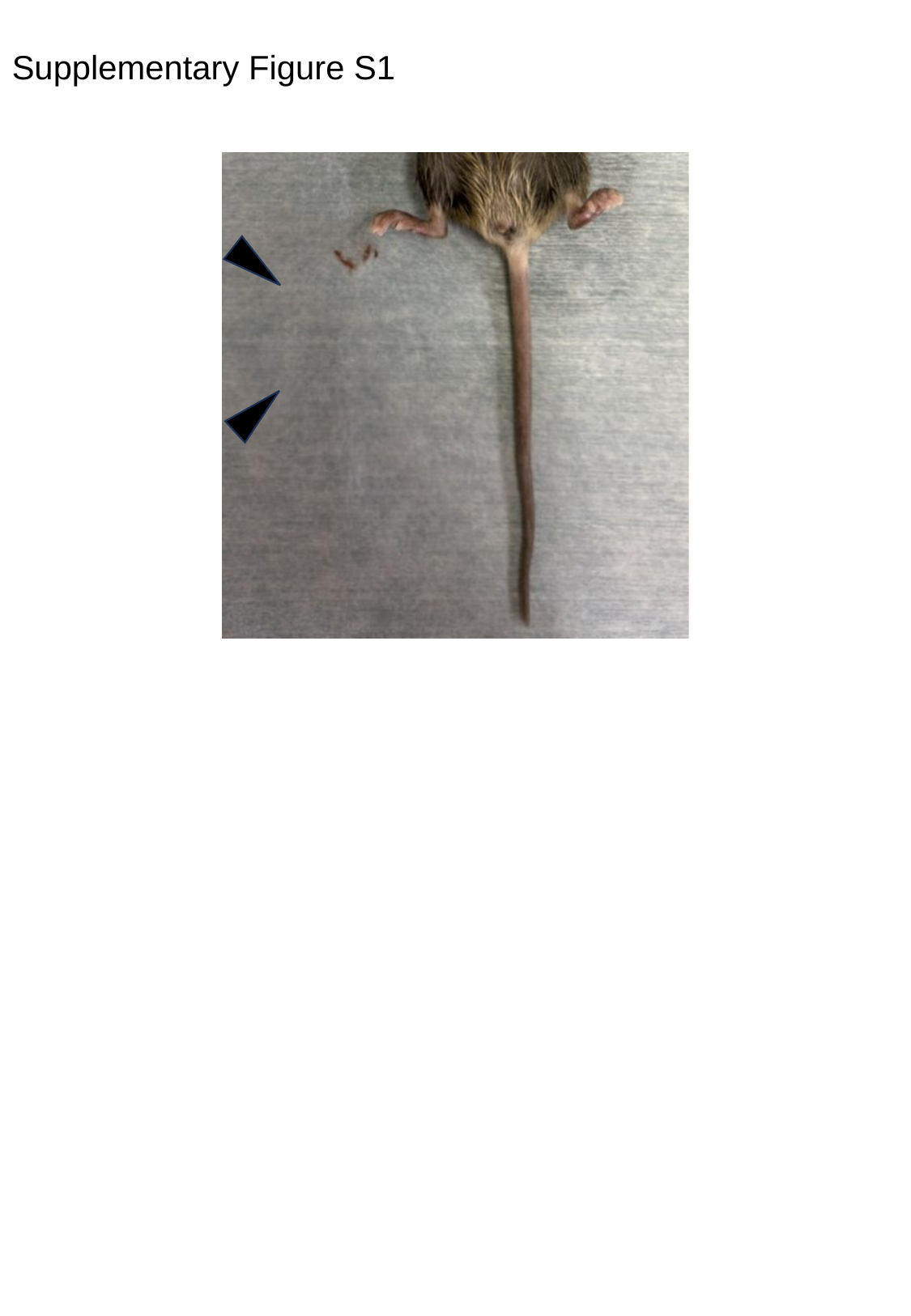

Supplementary Figure S1

## Slide 2
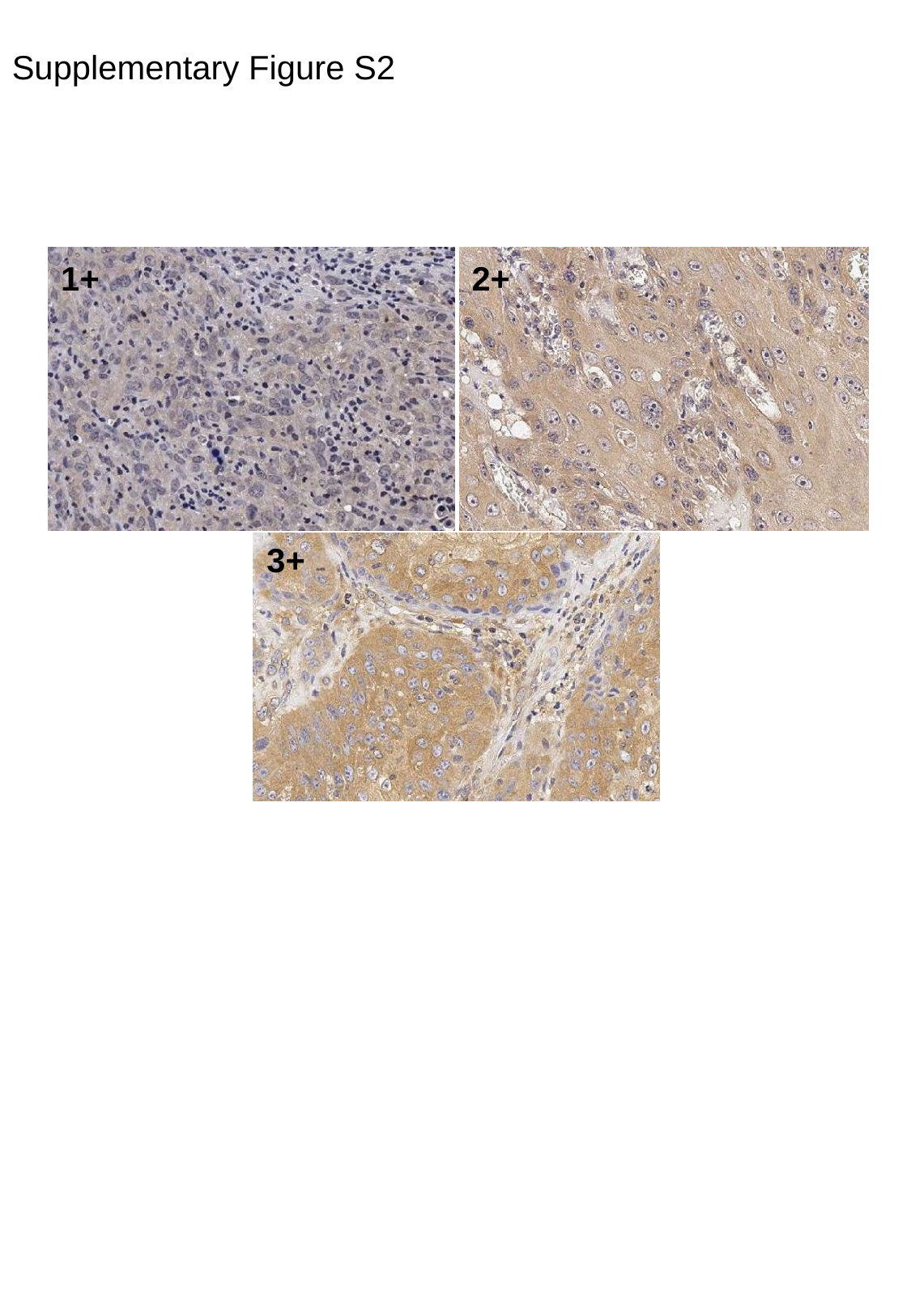

Supplementary Figure S2
1+
2+
3+

## Slide 3
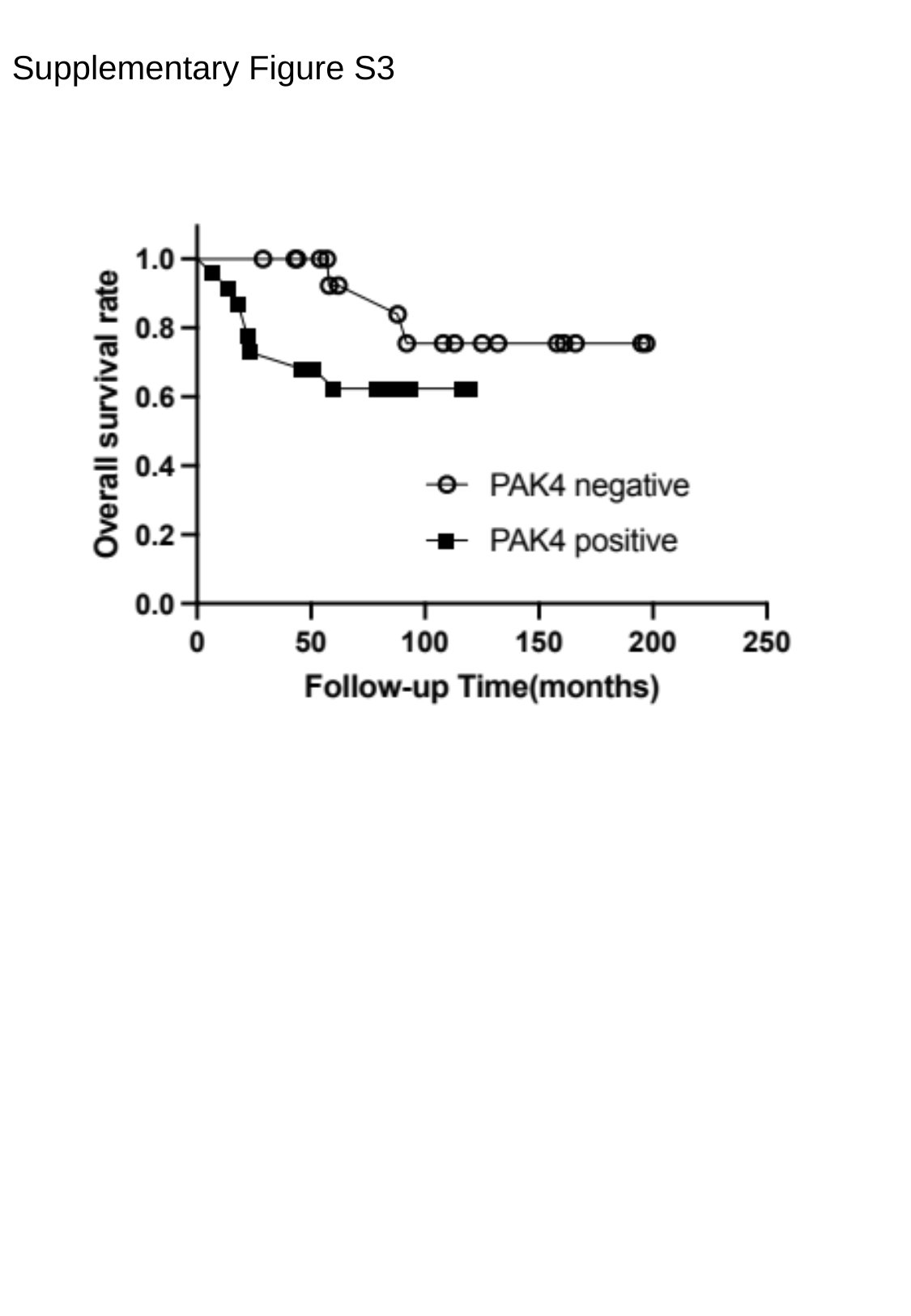

Supplementary Figure S3

## Slide 4
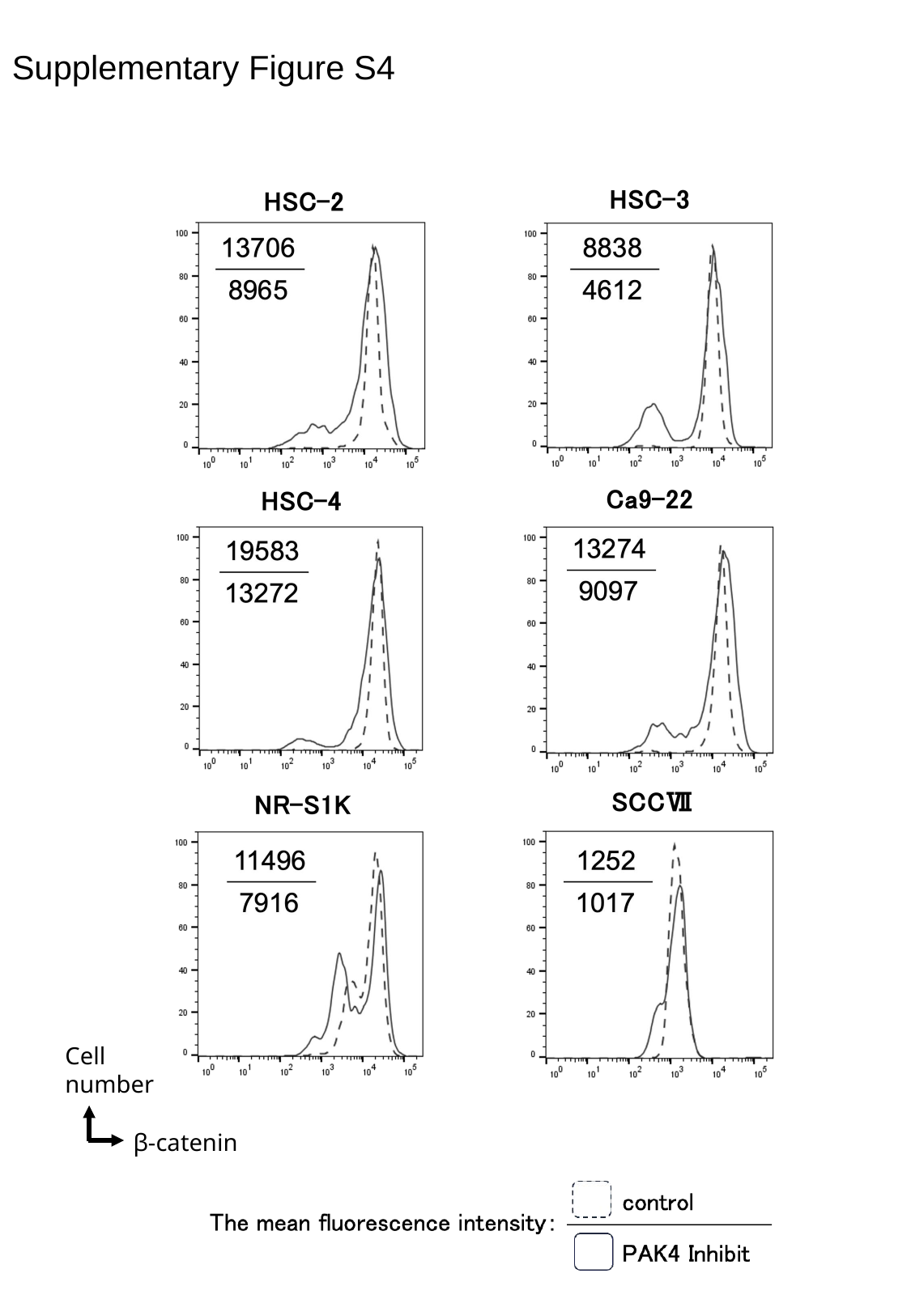

Supplementary Figure S4
Cell
number
β-catenin

## Slide 5
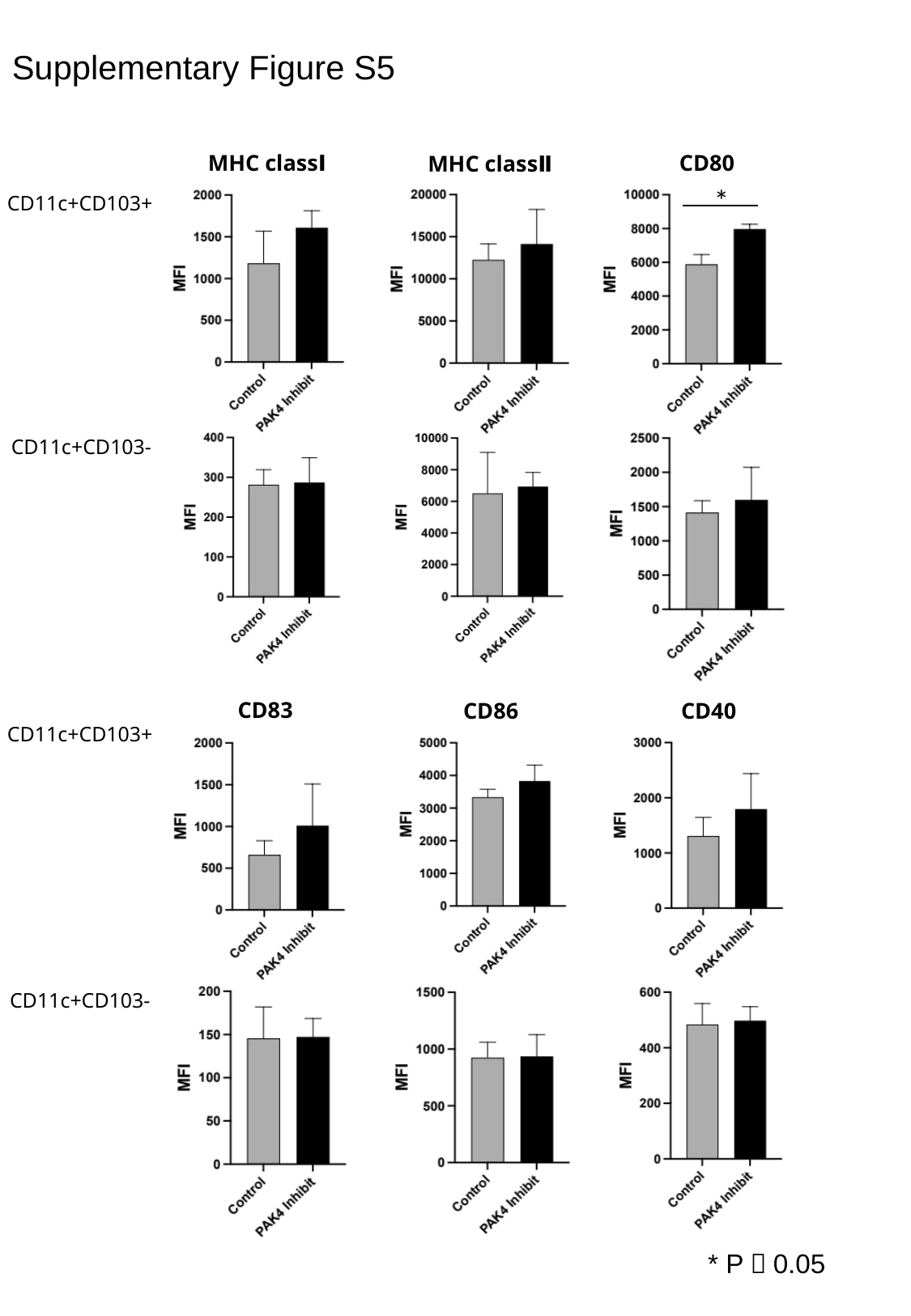

Supplementary Figure S5
MHC classⅠ
CD80
MHC classⅡ
*
CD11c+CD103+
CD11c+CD103-
CD83
CD40
CD86
CD11c+CD103+
CD11c+CD103-
* P＜0.05
