# Supplementary material for: PAK4 inhibition augments anti-tumour effect by immunomodulation in oral squamous cell carcinoma
Source: Sci Rep. 2024 Jun 18;14:14092. doi: 10.1038/s41598-024-64126-0 (PMC11189426; doi:10.1038/s41598-024-64126-0)
Supplement: Supplementary file 2 — Supplementary Legends. [file 41598_2024_64126_MOESM2_ESM.docx]

**Supplementary Figure S1.**

**A mouse xenograft model of oral squamous cell carcinoma.**

After the inoculation of tumours into the right masseter of C3H/HeN mice, the subcutaneous tumour area (length × width) was measured every two days using a calliper.

**Supplementary Figure S2.**

**Images depicting the various degrees of PAK4 staining.**

The staining intensity for each sample was designated as follows: weak (1+), moderate (2+), and strong (3+).

**Supplementary Figure S3.**

**Kaplan–Meier curves for overall survival rates of patients with OSCC negative or positive for PAK4 protein.**

Patients with positive PAK4 expression in tumours tended toward a poorer prognosis than those with negative expression, though the observed difference did not achieve statistical significance (P = 0.094, log-rank test).

**Supplementary Figure S4.**

**Alterations in β-catenin expression caused by PAK4 inhibitor treatment in oral squamous cell carcinoma (OSCC) cell lines.**

Human OSCC cell lines, HSC-2, HSC-3, HSC-4, and Ca9-22, and mouse OSCC cell lines, SCCVII and NR-S1K, were cultured in the presence or absence of PF-3758309 for 48 h. Intracellular staining was performed and the expression of β-catenin was analysed using flow cytometry. Representative histograms are shown (*n* = 3/group). Numbers in each panel indicate the mean fluorescence intensity for each molecule in the control (upper value) and PAK4 inhibitor-treated (lower value) groups.

**Supplementary Figure S5.**

**Statistical analysis of phenotypic alterations in tumour-infiltrated dendritic cells (DCs) following administration of PAK4 Inhibitor in oral squamous cell carcinoma (OSCC) tumour-bearing mice.**

The bar graph presenting overall results and the statistical analysis results of the cell-surface expression of various lymphocyte co-stimulatory molecules on CD11c^+^CD103^−^ and CD11c^+^CD103^+^ cells are shown (n = 4/group); p < 0.05, control vs PF-3758309. The MFI of each stained molecule on CD11c^+^CD103^−^ and CD11c^+^CD103^+^ cells is shown.
